# IL-4 enhances IL-10 production in Th1 cells: implications for Th1 and Th2 regulation

**DOI:** 10.1038/s41598-017-11803-y

**Published:** 2017-09-12

**Authors:** Ruth E. Mitchell, Masriana Hassan, Bronwen R. Burton, Graham Britton, Elaine V. Hill, Johan Verhagen, David C. Wraith

**Affiliations:** 1 School of Cellular and Molecular Medicine, University of Bristol, Bristol, United Kingdom; 2 Present Address: Integrative Epidemiology Unit, School of Social and Community Medicine, University of Bristol, Bristol, BS8 2BN United Kingdom; 3 Present Address: Department of Pathology, Faculty of Medicine and Health Sciences, University Putra Malaysia, 43400 Serdang, Selangor Malaysia; 4 Present Address: Icahn School of Medicine at Mount Sinai, New York, USA; 5 Present Address: Department of Immunobiology, King’s College London School of Medicine, London, United Kingdom; 6 Present Address: Institute of Immunology and Immunotherapy, University of Birmingham, Birmingham, B15 2TT United Kingdom

## Abstract

IL-10 is an immunomodulatory cytokine with a critical role in limiting inflammation in immune-mediated pathologies. The mechanisms leading to IL-10 expression by CD4^+^ T cells are being elucidated, with several cytokines implicated. We explored the effect of IL-4 on the natural phenomenon of IL-10 production by a chronically stimulated antigen-specific population of differentiated Th1 cells. *In vitro*, IL-4 blockade inhibited while addition of exogenous IL-4 to Th1 cultures enhanced IL-10 production. In the *in vivo* setting of peptide immunotherapy leading to a chronically stimulated Th1 phenotype, lack of IL-4Rα inhibited the induction of IL-10. Exploring the interplay of Th1 and Th2 cells through co-culture, Th2-derived IL-4 promoted IL-10 expression by Th1 cultures, reducing their pathogenicity *in vivo*. Co-culture led to upregulated c-Maf expression with no decrease in the proportion of T-bet^+^ cells in these cultures. Addition of IL-4 also reduced the encephalitogenic capacity of Th1 cultures. These data demonstrate that IL-4 contributes to IL-10 production and that Th2 cells modulate Th1 cultures towards a self-regulatory phenotype, contributing to the cross-regulation of Th1 and Th2 cells. These findings are important in the context of Th1 driven diseases since they reveal how the Th1 phenotype and function can be modulated by IL-4.

## Introduction

The immunomodulatory cytokine, interleukin-10 (IL-10), plays a crucial role in the maintenance of homeostasis and in particular in controlling immunopathology in infectious, allergic and autoimmune diseases^1^. The majority of cells in both the innate and adaptive immune system can express IL-10, including multiple subsets of T cells, consistent with the concept of plasticity^2^. The natural phenomenon of IL-10 secretion by chronically stimulated T helper 1 (Th1) cells is important in the regulatory phenotype of these effector cells: this anti-inflammatory cytokine limits the immune response to both self- and non-self antigens, protecting the host from excessive Th1 pathology and preventing tissue damage^3^. This phenomenon occurs in the context of infection^4, 5^ as well as in auto-inflammatory diseases such as colitis^6^ and autoimmune conditions including rheumatoid arthritis^7^.

Indeed, in the Tg4 T cell receptor transgenic mouse model, administration of the Ac1-9 peptide of myelin basic protein (MBP) induces a Th1 skewed environment characterised by interferon-γ (IFN-γ) positive and T-bet positive cells. In this environment, repetitive antigen administration induced IL-10 production from T cells and protected mice from experimental autoimmune encephalomyelitis (EAE)^8, 9^. IL-10 renders dendritic cells less effective at priming CD4^+^ T cells thus creating a negative feedback loop suppressing further differentiation of Th1 cells and preventing excessive Th1 inflammation^8^.

The induction of IL-10 is, therefore, critical for the control of Th1-mediated immune pathology and the molecular events that regulate its induction become important potential therapeutic targets. Several cytokines increase IL-10 expression by Th1 cells, notably IL-12^10^, IL-27^11, 12^ and IL-21^13^. Interleukin-4 (IL-4) has also been shown to cause upregulation of IL-10 production in LPS stimulated macrophages^14^.

In this study, we investigated whether IL-4 induces IL-10 expression from a population of previously differentiated Th1 cells both *in vitro* and *in vivo* through repeated stimulation of Tg4 CD4^+^ T cells. *In vitro*, both the IL-4 produced by the population of Th1 cells themselves and exogenous IL-4 led to an increase in IL-10 production among IFN-γ^+^ and T-bet^+^ cultures. The lack of IL-4Rα inhibited the induction of IL-10 in the context of repeated stimulation *in vivo*. This led to examination of the interaction of Th1 and T helper Th2 (Th2) cells showing that IL-4 secreted by Th2 cells upregulated IL-10 production in Th1 cultures which correlated with an induction of the transcription factor c-Maf. Furthermore, IL-4 dampened the pathogenicity of encephalitogenic T cells in adoptively transferred EAE. We conclude that IL-4 induces IL-10 from CD4^+^ T cells thereby controlling the balance of pro- and anti-inflammatory cytokines and promoting a Th1 regulatory (Tr1-like) phenotype.

## Results

### Repeated stimulation of Th1 cells induces the production of IL-10

**Figure 1:**
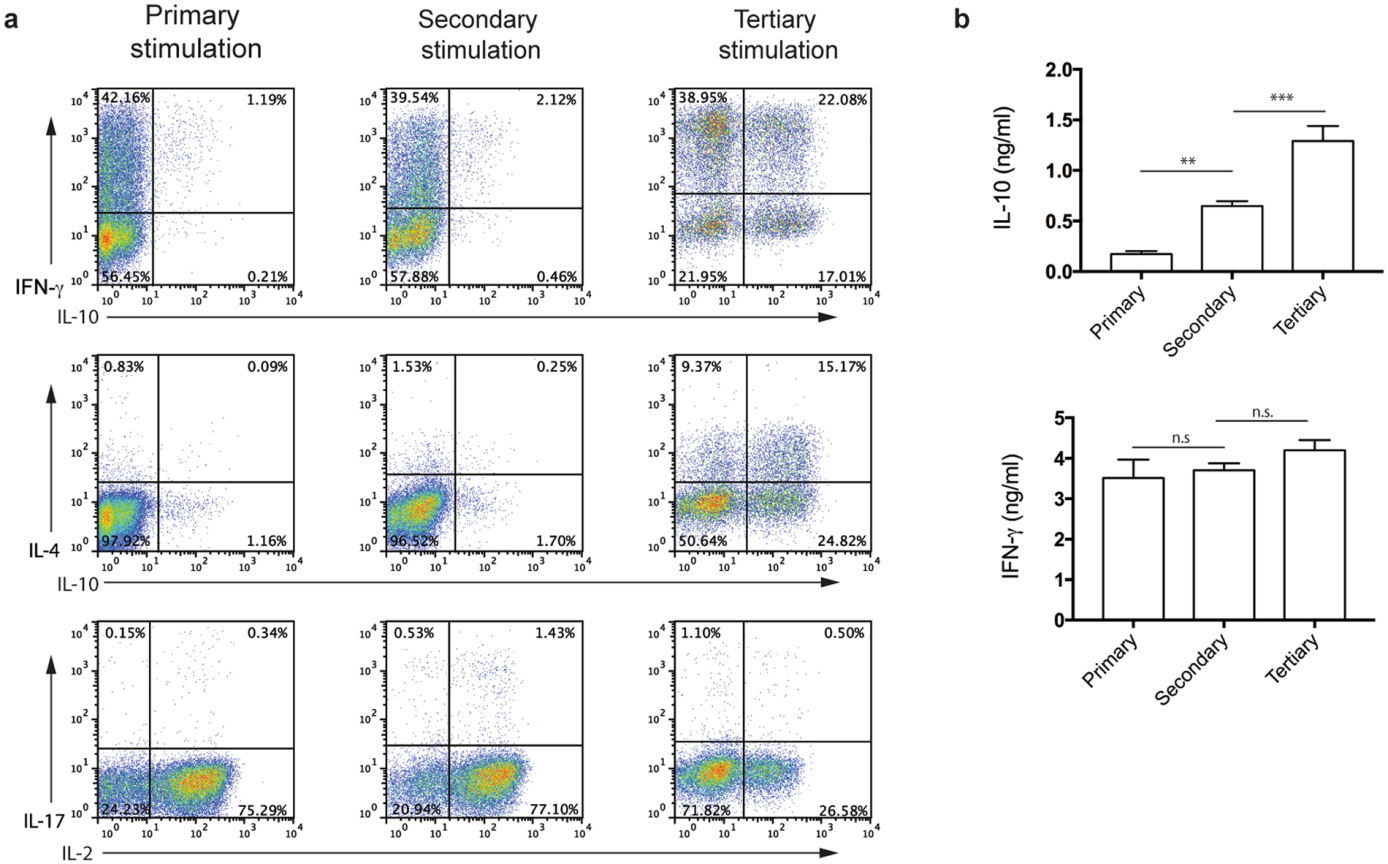
Chronic antigen stimulation leads to IL-10 production. (**a**) Splenic Tg4 CD4^+^ cells were cultured with irradiated B10.PL splenocytes as APCs and 10 μg/ml of MBP Ac1-9[4K] peptide (Primary stimulation). After 7 days, viable cells were restimulated with irradiated B10.PL splenocytes as APCs, MBP Ac1-9[4K] peptide (Secondary stimulation). This was repeated for a third cycle (Tertiary stimulation). Intracellular cytokine staining for IFN-γ, IL-10, IL-4, IL-17, IL-2, was carried out on day 7 of each cycle of stimulation following PMA and ionomycin stimulation. Data are plots gated on live Vβ8^+^ cells and are representative of three independent experiments. (**b**) Polarised Th1 cells (Primary stimulation) were stimulated for a further two cycles of 7 days each (Secondary and Tertiary stimulations) with irradiated B10.PL splenocytes as APCs in the presence of MBP Ac1-9[4K]. Tissue culture supernatants were taken on day 7 of each stimulation and analysed for IL-10 and IFN-γ by ELISA. Data are mean + SD from triplicate wells from one experiment, representative of two independent experiments. n.s. (non-significant) p > 0.05, **p < 0.01, ***p < 0.001, (ANOVA, Tukey’s multiple-comparison post-test).

To mirror the induction of IL-10 observed *in vivo* following repeated administration of MBP^15^, Tg4 splenocytes, expressing a transgenic TCR specific for the N-terminal peptide Ac1-9 of MBP, were stimulated with cognate peptide for a total of three stimulations (Tertiary stimulation). These cells did not show any change in IFN-γ expression although there was a decrease in the percentage of IL-2 cells (Fig. 1a). Both the amount populations of IL-10^+^ and IL-4^+^ T cells increased after each round of stimulation. We sought to understand if the observed increase in IL-10 also occurred following Th1 cell polarisation. We restimulated a population of differentiated Th1 cells *in vitro* as has previously been described^10, 16, 17^. Tg4 splenocytes, were differentiated into Th1 cells in the presence of IL-12 and anti-IL-4 and were subsequently stimulated a further two times with antigen-presenting cells (APCs) and MBP peptide. IL-10 secretion significantly increased after each stimulation (Fig. 1b) whilst the production of IFN-γ remained unaltered suggesting that these cells maintain their Th1 phenotype.

### IL-4 inhibition leads to increased IL-10 production by Th1 cells

The cognate peptide MBP Ac1-9[4K] forms highly unstable complexes with the MHC class II molecule H-2 A^u^ ^18^. Comparing MBP Ac1-9 peptide analogs with an alanine or tyrosine substitution at position four, displaying a hierarchy of affinities for H-2 A^u^ (Ac1-9[4K] ≪ [4 A] ≪ [4Y]), the highest affinity peptide, Ac1-9[4Y], was the most potent stimulus for IL-10 induction *in vivo* following repeated administration^19^. These MBP Ac1-9 analogs were used to stimulate CD4^+^ T cells to investigate IL-10 production *in vitro*. The highest affinity peptide, MBP Ac1-9[4Y] was used in all subsequent experiments to ensure a consistently high proportion of IL-10 expressing cells (see Supplementary Fig. S2).

**Figure 2:**
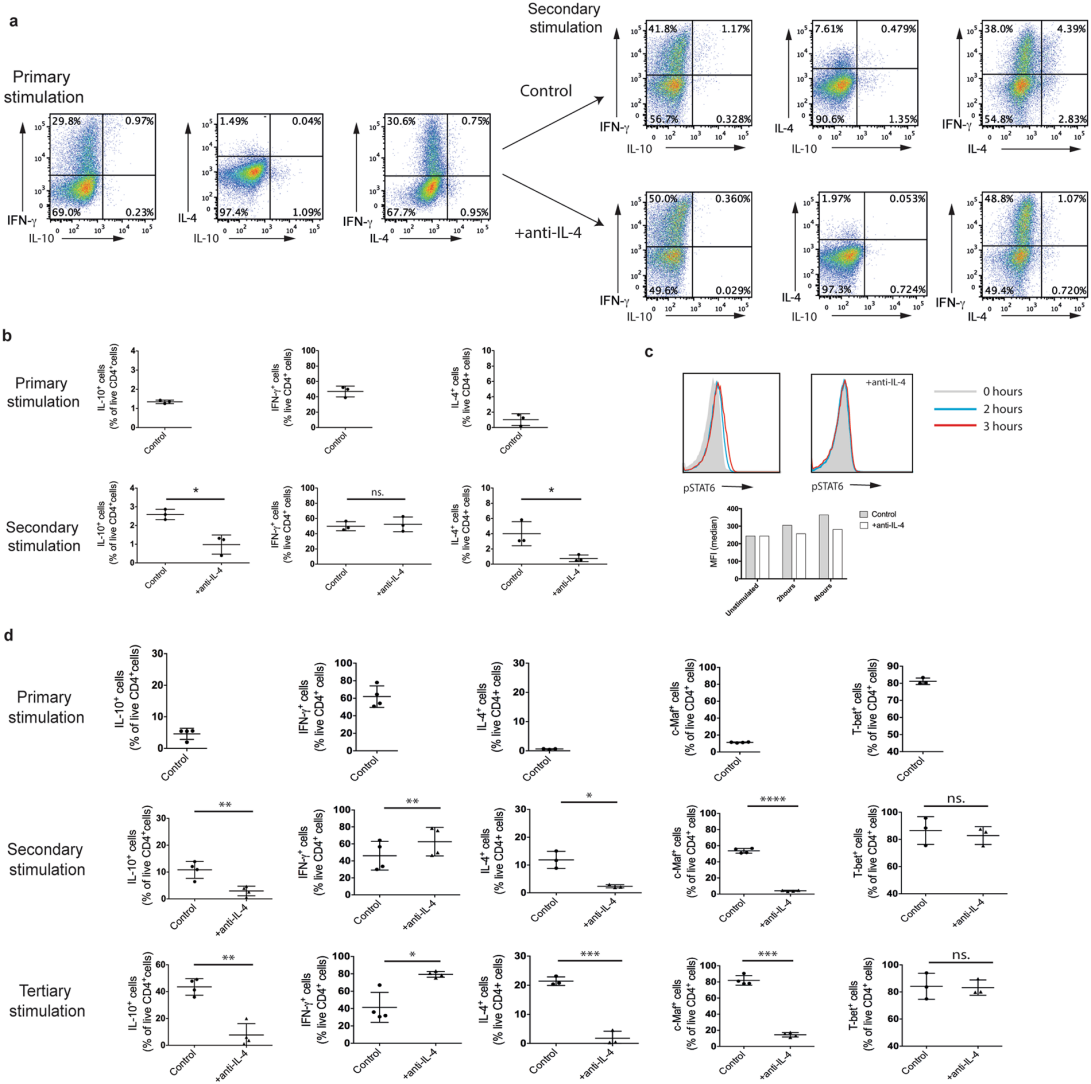
IL-4 regulates IL-10 production in polarised Th1 cells. (**a**,**b**) Polarised Tg4 Th1 cells (Primary stimulation) were stimulated for a further cycle (Secondary stimulation) with irradiated B10.PL splenocytes APCs with MBP Ac1-9[4Y] in the presence of IL-12 (Control) or IL-12 and anti-IL-4 blocking antibody (+anti-IL-4). Intracellular cytokine staining was carried out on day 7 of each stimulation following PMA and ionomycin stimulation. (**a**) Representative FACS plots of three independent experiments are gated on live CD4^+^ cells and show IFN-γ, IL-4 and IL-10 expression. (**b**) Pooled quantitation of IL-10^+^, IFN-γ^+^ and IL-4^+^ live CD4^+^ cells from three independent experiments as shown in (**a**). Error bars are mean ± SEM. n.s (not significant) p > 0.05, *p ≤ 0.05 (Student’s paired t-test). (**c**) Polarised CD4^+^ selected Th1 cells were stimulated with anti-CD3 and anti-CD28 for 2, 3 and 4 hours in a time course with (+anti-IL-4) or without anti-IL-4 blocking antibody. Histograms of phospho-STAT6 staining gated on live CD4^+^ cells and graphs of geometric mean fluorescence are shown and are representative of two independent experiments. (**d**) Polarised CD4^+^ selected Th1 cells (Primary stimulation) were stimulated for a further two cycles of 7 days each (Secondary and Tertiary stimulation) with anti-CD3 and anti-CD28 in the presence of IL-12 (Control) or IL-12 and anti-IL-4 blocking antibody (+anti-IL-4). Intracellular cytokine staining for IFN-γ, IL-4 and IL-10 was carried out on day 7 of each cycle of stimulation following PMA and ionomycin stimulation. Graphs show pooled quantitation of IL-10^+^, IFN-γ^+^ and IL-4^+^ live CD4^+^ cells from three independent experiments. Error bars are mean ± SEM. n.s (not significant) p > 0.05, *p ≤ 0.05, **p ≤ 0.01, ***p < 0.001, ****p ≤ 0.0001 (Student’s paired t-test).

To investigate whether the increase in IL-4 observed following stimulation of Th1 cells cultures was sufficient to cause an upregulation of IL-10 (Fig. 1), a population of polarised Th1 cells were stimulated with APCs and peptide in the presence of an anti-IL-4 blocking antibody. IL-4 is known to promote its own expression^20^ and as expected, when IL-4 was blocked, the proportion of IL-4^+^ CD4^+^ T cells was decreased. This was concomitant with a significant decrease in IL-10^+^ cells (Fig. 2a and b) and no change in IFN-γ expression (Fig. 2b). Blocking IL-4 inhibited the time-dependent phosphorylation of Signal Transducer and Activator of Transcription 6 (STAT6), a Jak-Stat kinase downstream of the IL-4 receptor^21^, in CD4 selected cells from the polarised Th1 cells cultures stimulated with anti-CD3/anti-CD28 (Fig. 2c). These findings suggest that upon a second stimulation, a small percentage of a population of polarised Th1 cells express IL-4 that acts in a population-based autocrine feedback loop to cause the upregulation of IL-10 through the phosphorylation of STAT6.

We next assessed the requirement of APCs during IL-4 mediated induction of IL-10 in a population of Th1 cells. CD4^+^ selected cells from this Th1 population were cultured with anti-CD3/anti-CD28 stimulation in the presence of anti-IL-4 blocking antibody for 7 days and then further stimulated under the same conditions (Fig. 2d). The increase in the percentage of IL-10^+^ and IL-4^+^ cells observed with each stimulation in these polarised Th1 cultures was significantly inhibited in the presence of anti-IL-4 blocking antibody (Fig. 2d). This indicates that IL-4 induced by subsequent stimulation acts directly on polarized CD4^+^ Th1 cultures increasing their IL-10 expression, suggestive of the self-regulatory phenotype of Th1 cells.

c-Maf regulates IL-10 production in macrophages and is correlated with IL-10 expression in CD4^+^ T cells^10, 14^. The blockade of IL-4 inhibited the increase in the transcription factor c-Maf observed following multiple stimulations of a population of Th1 cells. This suggests that c-Maf is a potential mechanism through which IL-4 regulates the upregulation of IL-10. The percentage of these cultures of polarised Th1 cells expressing T-bet, the master transcription factor of Th1 cells^22^, was unchanged over the course of subsequent stimulations as well as in the presence of anti-IL-4 showing a maintenance of their Th1 phenotype.

### Lack of IL-10 induction in IL-4Rα^−/−^ mice

**Figure 3:**
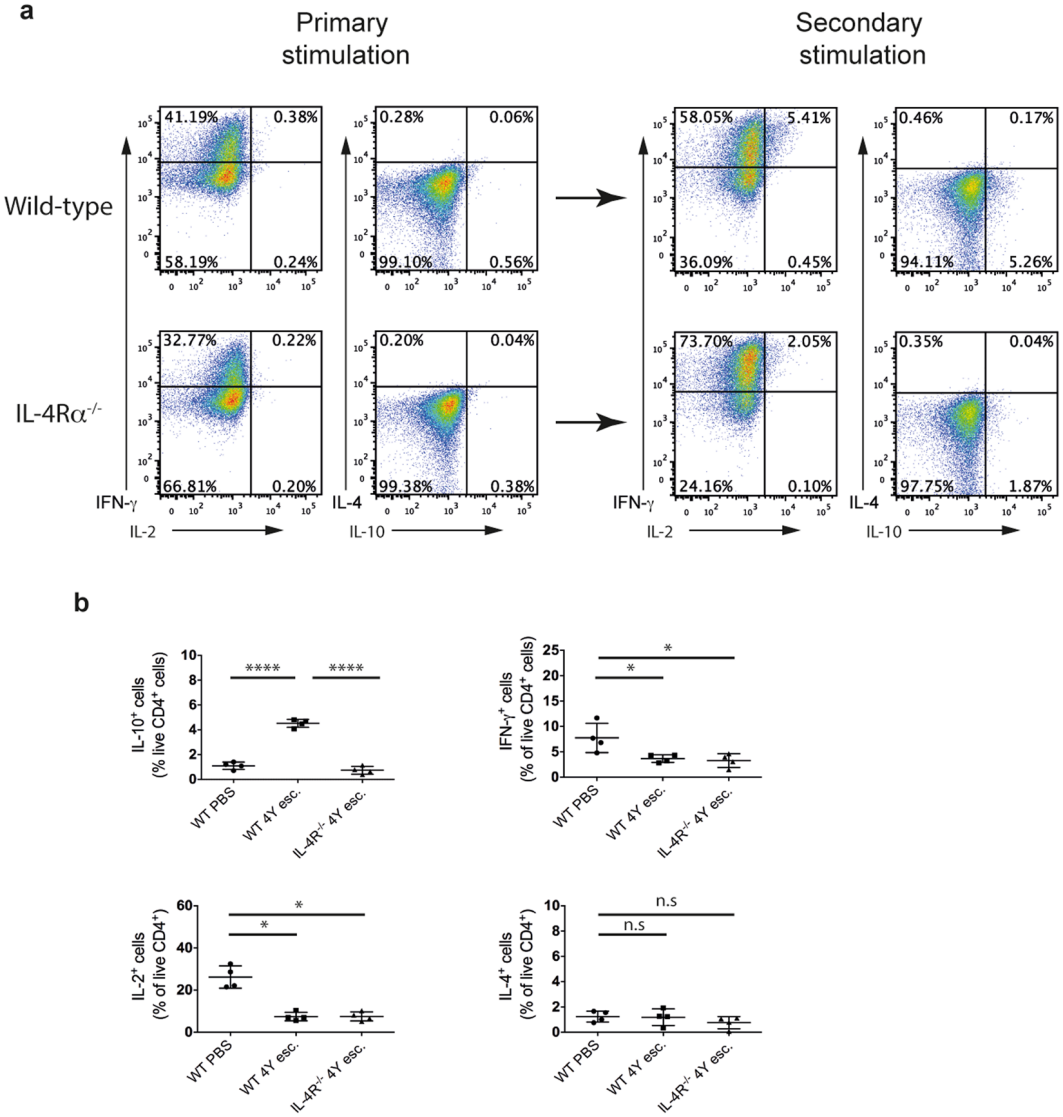
IL-4Rα deficiency inhibits the induction of IL-10. (**a**) Polarised Th1 cells from WT Tg4 and IL-4Rα^−/−^ Tg4 splenocytes (Primary stimulation) were stimulated for a further cycle (Secondary stimulation) with APCs and MBP Ac1-9[4Y] in the presence of IL-12. Intracellular cytokine staining was carried out on day 7 of each stimulation following PMA and ionomycin stimulation. Representative FACS plots of two independent experiments are gated on live CD4^+^ cells and show IFN-γ, IL-4 and IL-10 expression. (**b**) Tg4 mice (WT) and Tg4 IL-4Rα^−/−^ mice were treated subcutaneously every three to four days with an escalating dose of MBP Ac1-9[4Y] (0.08 μg, 0.8 μg, 8 μg, 80 μg, 80 μg, 80 μg) (4Y esc.) or with PBS as a control. Spleens were harvested two hours after the last treatment and intracellular cytokine staining was performed for IL-10, IL-4, IL-2 and IFN-γ following PMA and ionomycin stimulation. Graphs show pooled data of IL-10^+^, IFN-γ^+^, IL-2^+^ and IL-4^+^ live CD4^+^ cells from a representative experiment, from two independent experiments, with three to four animals in each treatment groups. Error bars are mean ± SEM. n.s (not significant) p > 0.05, *p ≤ 0.05, ****p ≤ 0.0001 (one-way ANOVA with Tukey’s multiple-comparison post-test).

Populations of Th1 cells differentiated from IL-4Rα^−/−^ splenocytes show a reduced but not completely abrogated induction of IL-10 expression following a second stimulation (Fig. 3a). To investigate whether IL-4 is responsible for the induction of IL-10 in an *in vivo* setting, a series of escalating doses of MBP Ac1-9[4Y] (0.08 μg, 0.8 μg, 8 μg, 80 μg, 80 μg, 80 μg) was administered subcutaneously to either Tg4 or IL-4Rα^−/−^ mice. This induced a population of CD4^+^ IL-10-expressing cells subsequent to an upregulation in T-bet and IFN-γ expression, indicating Th1 pathology^8, 15^ (Fig. 3b). The lack of IL-4Rα inhibited the induction of IL-10 expression. Peptide treatment *in vivo* significantly reduced the expression of IFN-γ and IL-2 compared to a single dose of peptide *in vitro*. There was no difference in expression of IFN-γ and IL-2 in CD4^+^ T cells in IL-4Rα^−/−^ animals (Fig. 3b). This suggests that in the context of peptide immunotherapy, IL-4 induces the expression of IL-10.

### IL-4 leads to increased IL-10 production by Th1 cells

**Figure 4:**
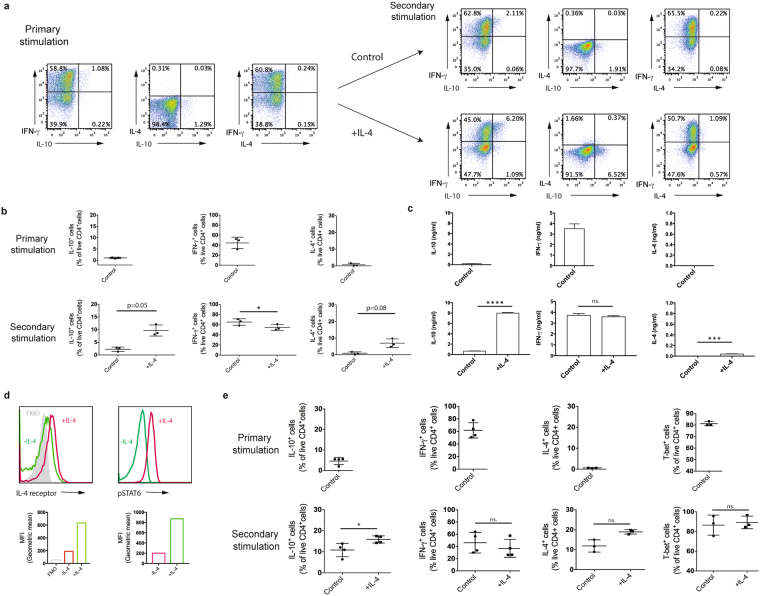
IL-4 induces IL-10 production in polarised Th1 cells. (**a**–**c**) Polarised Tg4 Th1 cells (Primary stimulation) were stimulated for a further cycle (Secondary stimulation) with irradiated B10.PL splenocytes APCs with MBP Ac1-9[4Y] in the presence of IL-12 (Control) or IL-12 and exogenous IL-4 (+IL-4). Intracellular cytokine staining was carried out on day 7 of each stimulation following PMA and ionomycin stimulation. (**a**) Representative FACS plots of three independent experiments are gated on live CD4^+^ cells and show IFN-γ, IL-4 and IL-10 expression. (**b**) Pooled quantitation of IL-10^+^, IFN-γ^+^ and IL-4^+^ live CD4^+^ cells from three independent experiments as shown in (**a**). Error bars are mean ± SEM. n.s (not significant) p > 0.05, *p ≤ 0.05 (Student’s paired t-test). (**c**) Tissue culture supernatants were taken on day 7 of each stimulation and analysed for IL-10, IFN-γ and IL-4 by ELISA. Data are mean + SD from triplicate wells from one experiment, representative of two independent experiments. n.s. (non-significant) p > 0.05, ***p < 0.001, ****p < 0.0001 (Student unpaired t-test). (**d**) Polarised CD4^+^ selected Th1 cells were stimulated with anti-CD3 for 20 minutes in the presence of IL-4. Histograms of IL-4 receptor and phospho-STAT6 staining gated on live CD4^+^ cells and graphs of geometric mean fluorescence are shown and are representative of two independent experiments. (**e**) Polarised CD4^+^ selected Th1 cells (Primary stimulation) were stimulated for a further cycle of 7 days (Secondary stimulation) with anti-CD3 and anti-CD28 in the presence of IL-12 (Control) or IL-12 and exogenous IL-4 (+IL-4). Intracellular cytokine staining for IFN-γ, IL-4 and IL-10 was carried out on day 7 of each cycle of stimulation following PMA and ionomycin stimulation. Graphs show pooled quantitation of IL-10^+^, IFN-γ^+^ and IL-4^+^ live CD4^+^ cells from four independent experiments. Error bars are mean ± SEM. n.s (not significant) p > 0.05, *p ≤ 0.05 (Student paired t-test).

We extended our studies by investigating the effect of exogenous IL-4 on a population of polarised Th1 cells. There was an increase in the percentage of cells expressing IL-10 with the addition of IL-4 following stimulation using APCs and peptide (Fig. 4a and b). This was reflected in a significant increase in IL-10 secretion (Fig. 4c). Although there was a slight decrease in IFN-γ expression in response to exogenous IL-4, this did not lead to a significant decrease in IFN-γ secretion from the population of Th1 cells following a further round of stimulation. Both the expression and secretion of IL-4 was increased in the presence of exogenous IL-4 (Fig. 4b and c).

The upregulation of IL-4 receptor and phosphorylation of STAT6 in CD4^+^ cells from the population of polarised Th1 cells in the presence of exogenous IL-4 together suggest that these mechanisms may be responsible for the downstream signalling (Fig. 4d).

To assess the requirement of APCs during IL-4 mediated induction of IL-10, polarised CD4^+^ selected from Th1 cultures were stimulated with anti-CD3/anti-CD28 (Fig. 4e). The presence of IL-4 caused a significant increase in the percentage of IL-10^+^ CD4^+^ cells with no significant differences in the proportion of IFN-γ^+^ or IL-4^+^ CD4^+^ T cells. This shows that exogenous IL-4 acts directly on a population of polarised Th1 cells to cause the induction of IL-10. T-bet was expressed by the majority of these cells (Fig. 4e) and the percentage of cells that expressed T-bet remained unchanged following a second round of stimulation and with the addition of IL-4. This suggests that the restimulated culture maintains their Th1 phenotype and the increase in IL-10 is not a deviation to another phenotype.

### Th2-derived IL-4 modulates Th1 cell phenotype by increasing their production of IL-10

**Figure 5:**
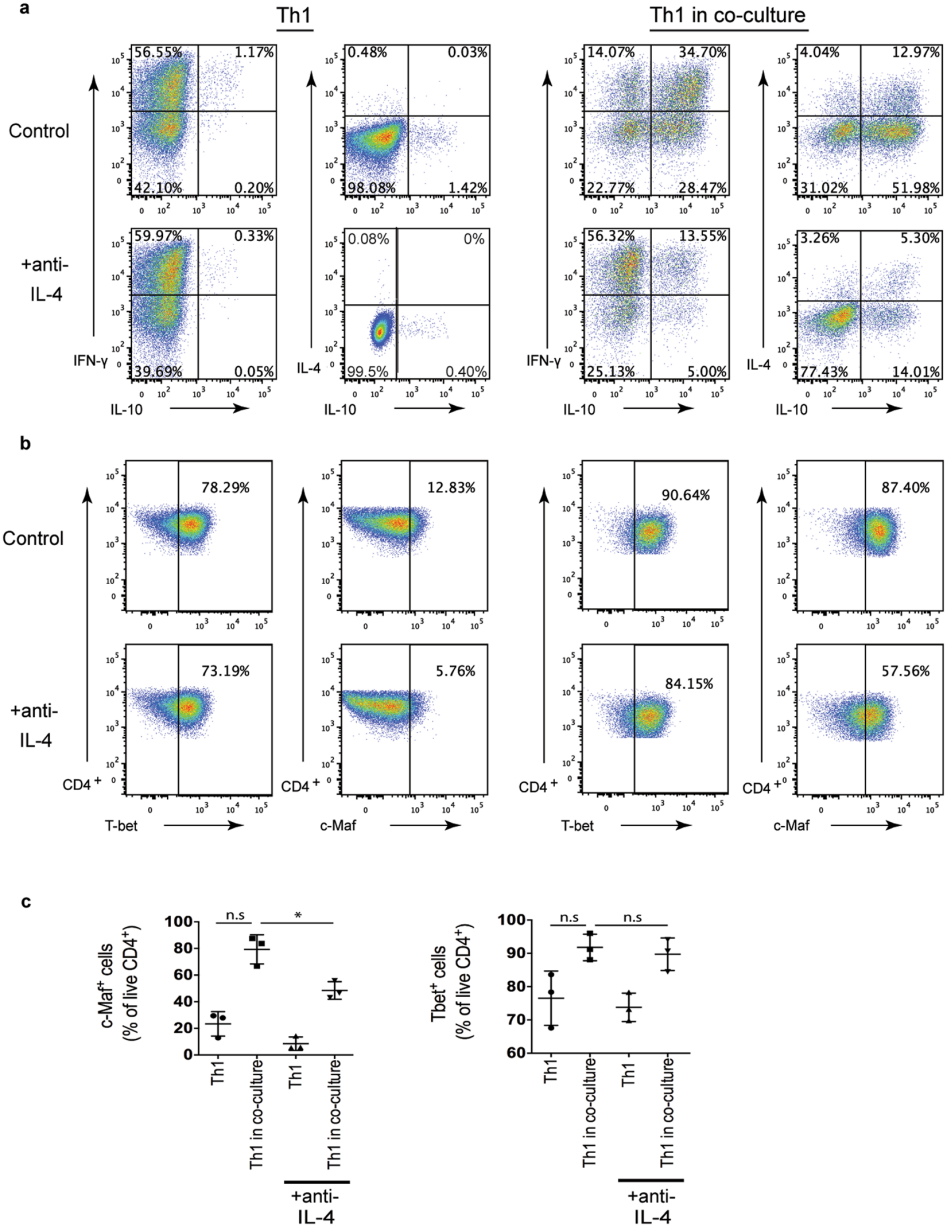
Th2-derived IL-4 induces IL-10 in Th1 cells. (**a**–**c**) Th1 and Th2 cells were polarised from CD45.1^+^ and CD45.2^+^ Tg4 splenocytes respectively for 7 days. Polarised Th1 cells were stimulated for a further cycle with irradiated B10.PL splenocytes APCs and MBP Ac1-9[4Y] either alone (Th1) or in co-culture with polarised Th2 cells (Th1 in co-culture) with (+anti-IL-4) or without anti-IL-4 blocking antibody (Control). (**a**) Intracellular cytokine staining was carried out on day 7 of the second stimulation, following PMA and ionomycin stimulation. Representative FACS plots of three independent experiments are gated on live CD4^+^ CD45.1^+^ (Th1) cells and show IFN-γ, IL-4 and IL-10 expression. (**b**) Transcription factor staining for T-bet and c-Maf was carried out on day 7 of the second stimulation. Representative FACS plots of three independent experiments are gated on live CD4^+^ CD45.1^+^ (Th1) cells and show T-bet and c-Maf expression. (**c**) Graphs show pooled quantitation of c-Maf^+^ and T-bet^+^ live CD4^+^ CD45.1^+^ cells from three independent experiments as shown in (**b**). Error bars are mean ± SEM. n.s (not significant) p > 0.05, *p ≤ 0.05 (ANOVA < Dunnett’s multiple-comparison post-test).

The IL-4 mediated induction of IL-10 in a population of polarised Th1 cells was further examined by using a population of cultured Th2 cells as an endogenous source of IL-4. This also provided the opportunity to investigate the *in vitro* interaction between these two differentiated T effector cell subsets. Polarised Th1 CD45.1^+^ Tg4 and Th2 CD45.2^+^ Tg4 cells were co-cultured with APCs and peptide. The presence of Th2 cells caused an increase in the percentage of IL-10 and IL-4 expressing cells in the Th1 culture. Blocking IL-4 during co-culture inhibited the upregulation of IL-10 and IL-4 expression by Th1 cultures (Fig. 5a). The presence of anti-IL-4 significantly inhibited the increase in the proportion of c-Maf^+^ cells in the population of Th1 cells observed following co-culture of Th1 and Th2 cells (Fig. 5c). This mirrored the trend in IL-10 expression across the different conditions, suggesting that c-Maf is a regulator of IL-10 expression in this setting. Interestingly, there was an increase in T-bet expression within the Th1 culture in the presence of Th2 cells and blocking IL-4 did not influence this. Therefore, Th2-derived IL-4 upregulates IL-10 and c-Maf expression in a population of majority T-bet^+^ cells.

**Figure 6:**
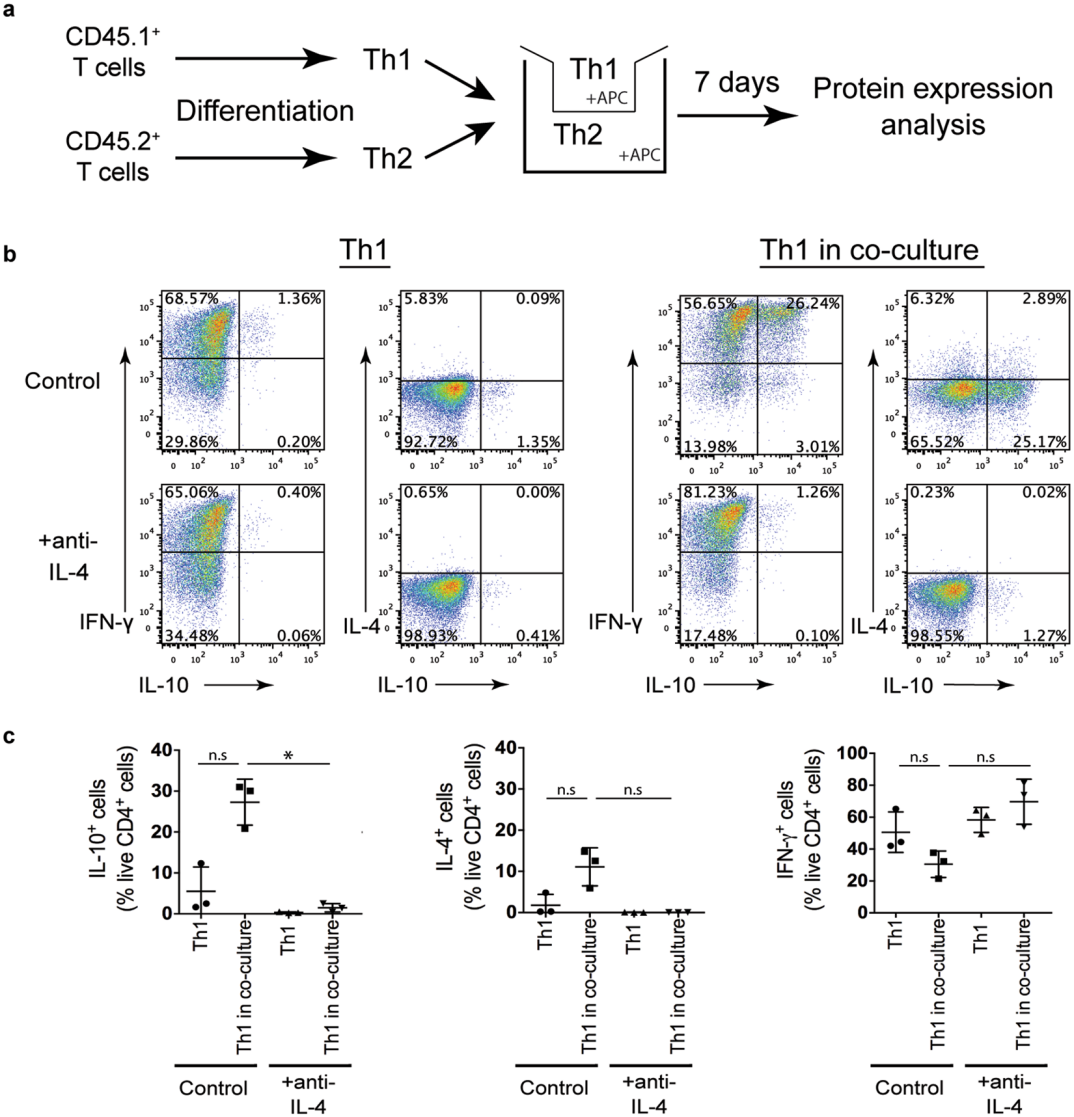
Secreted Th2 derived IL-4 increases production of IL-10 in Th1 cells. Th1 and Th2 cells were polarised from CD45.1^+^ and CD45.2^+^ Tg4 splenocytes respectively for 7 days. Polarised Th1 cells were stimulated for a further cycle with irradiated B10.PL splenocytes as APCs and MBP Ac1-9[4Y] either alone (Th1) or in the presence of polarised Th2 cells separated by a transwell membrane (Th1 in co-culture) with (+anti-IL-4) or without anti-IL-4 blocking antibody (Control). (**a**) Schematic of transwell assay. (**b**) Intracellular cytokine staining was carried out on day 7 of the second stimulation, following PMA and ionomycin stimulation. Representative FACS plots of three independent experiments are gated on live CD4^+^ CD45.1^+^ (Th1) cells and show IFN-γ, IL-4 and IL-10 expression. (**c**) Graphs show pooled quantitation of IL-10^+^, IFN-γ^+^ and IL-4^+^ live CD4^+^ CD45.1^+^ (Th1) cells from three independent experiments. Error bars are mean ± SEM. n.s (not significant) p > 0.05, *p ≤ 0.05, **p ≤ 0.01 (ANOVA < Dunnett’s multiple-comparison post-test).

To determine whether contact between the Th1 and Th2 cells was required for upregulation of IL-10 in the Th1 culture, a transwell assay was performed. This enabled the separation of the T helper cell subsets via a semi-permeable membrane (illustrated in Fig. 6a). Splenocytes polarised into Th1 and Th2 cells were stimulated with peptide and APCs placed on either side of the transwell. Under control conditions, the proportion of the Th1 culture expressing IL-10 increased in a transwell co-culture with Th2 cells (Fig. 6b and c). Blocking IL-4 inhibited the induction of IL-10 expressing cells in the Th1 population. This correlated with complete inhibition of upregulation of IL-4 in these cells. This demonstrates that Th2-derived IL-4 induced IL-10 expression in Th1 cultures via a secreted mechanism. The trend towards a decrease in the proportion of IFN-γ^+^ cells in the presence of Th2 cells was inhibited when IL-4 was blocked.

### Th2-derived IL-4 reduces the pathogenicity of Th1 cells

**Figure 7:**
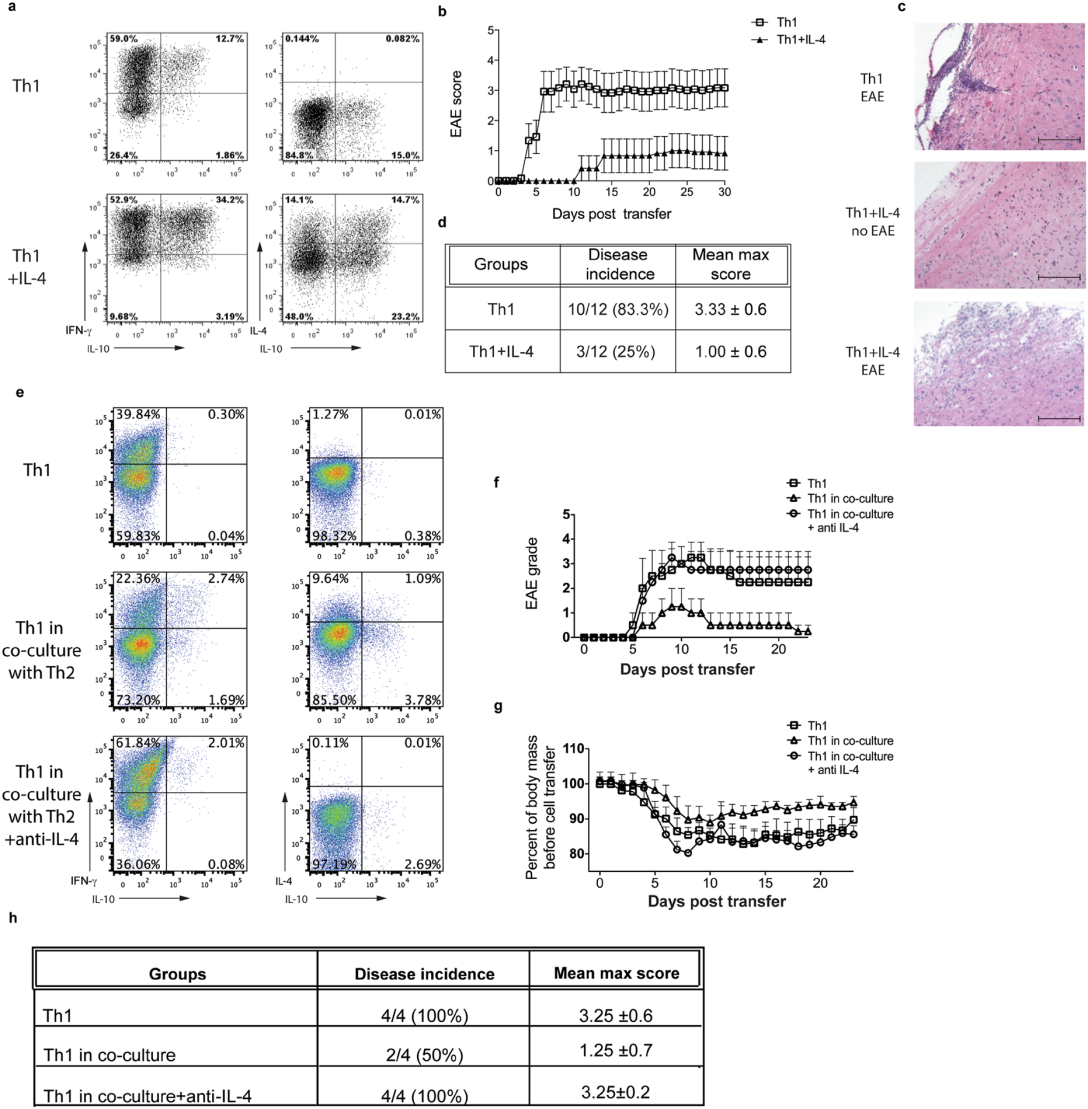
Th2 derived IL-4 reduces encephalogenic capacity of Th1 cells. (**a**,**b**) Polarised Th1 cells (Primary stimulation) were taken through a Secondary and Third stimulation with irradiated B10.PL splenocytes as APCs and MBP Ac1-9[4K] in the presence of IL-12 (Control) or IL-12 and exogenous IL-4 (Th1 + IL-4). On day 5 of the third stimulation, 1 × 10^7^ live Th1 cells were adoptively transferred to Tg4 mice by intraperitoneal injection (n = 4 per condition). Three independent experiments were performed. (**a**) Intracellular cytokine staining was carried out prior to transfer, following PMA and ionomycin stimulation. Representative FACS plots are gated on live CD4^+^ cells and show IFN-γ, IL-4 and IL-10 expression. (**b**) Time course of disease progression. EAE was scored daily. Graph shows mean EAE score ± SEM from each day. (**c**) Representative H&E staining at ×10 magnification of the spinal cord of mice in the following conditions: control Th1 cells (top) and Th1 cells + IL-4 with no disease score (middle) and Th1 cells + IL-4 with disease (bottom) (Bar = 750 µm). (**d**) Table displays key EAE factors: disease incidence is presented as diseased mice/total mice (percentage of diseased mice); mean maximum score as mean ± SEM. (**e–h**) Th1 and Th2 cells were polarised from CD45.1^+^ and CD45.2^+^ Tg4 splenocytes respectively. Polarised Th1 cells were stimulated for a further cycle with irradiated B10.PL splenocytes as APCs and MBP Ac1-9[4Y] either alone (Th1) or in the presence of polarised Th2 cells separated by a transwell membrane (Th1 in co-culture) with (+anti-IL-4) or without anti-IL-4 blocking antibody (Control). On day 4 of the second stimulation, 1 × 10^7^ Th1 cells were adoptively transferred to Tg4 mice by intraperiotineal injection (n = 4 per condition). EAE was scored and body mass recorded daily. Two similar experiments were performed. (**e**) Intracellular cytokine staining was carried out prior to transfer, following PMA and ionomycin stimulation. Representative FACS plots are gated on live CD4^+^ CD45.1^+^ (Th1) cells and show IFN-γ, IL-4 and IL-10 expression. (**f**) Time course of disease progression. Graph shows mean EAE score + SEM from each day. (**g**) Graph shows mean percentage weight loss, relative to initial body weight. (**h**) Table displays key EAE factors: disease incidence is presented as diseased mice/total mice; mean maximum score as mean + SEM.

IL-10 plays a protective role in EAE induced via antigen and CFA administration^23^ and also via adoptive T cell transfer^17^. We, therefore, addressed the biological relevance of the IL-4 induced upregulation of IL-10 in Th1 cultures investigating whether these cells would be less encephalitogenic in an *in vivo* setting. Following the tertiary round of stimulation, Th1 cultures in the presence of exogenous IL-4 resulted an increase in IL-10 expression (Fig. 7a). These were adoptively transferred to Tg4 mice to induce EAE. Culture of Th1 cells with IL-4 resulted in a later disease onset, a decrease in EAE severity that was sustained for the entire disease course as well as less disease incidence (Fig. 7b and d) and was accompanied by less infiltrating cells as compared to EAE induced by a control Th1 culture (Fig. 7c). Mice transferred with Th1 cells cultured alone have a histological score of grade 3 showing a series of mononuclear perivascular cuffs occurred in the meninges of the ventral hindbrain and these extended into adjacent white matter. Furthermore, local demyelination was observed which correlated with inflammation of peripheral white matter in the spinal cord. In addition, some small mononuclear cuffs appeared in deeper white matter (Fig. 7c top). Conversely, spinal cords from animals transferred with Th1 cells cultured in the presence of IL-4 have a grade of 0 in all samples with no disease score (Fig. 7c middle). For the few animals in this group in which disease occurred, the spinal cord revealed narrow plaques of lymphocytic to granulomatous inflammation which were associated with demyelination at the margins of peripheral white matter scoring 3. However, this correlated with less infiltration of inflammatory cells as compared to EAE induced by a control Th1 culture (Fig. 7c bottom).

We furthered this by investigating the effect of a population of differentiated Th2 cells on the encephalitogenic capacity of a population of Th1 cells, through secreted IL-4. Th1 cells that were co-cultured Th2 cells showed a slight increase in IL-10 expression compared to Th1 alone and this was accompanied by a decrease in EAE severity following adoptive transfer (Fig. 7e and f). This was reflected in a decreased weight loss (Fig. 7g) as well as a reduced mean max disease score (Fig. 7h) suggesting that IL-4 reduces the pathogenicity of Th1 cultures. The day of onset was the same for both the Th1 culture and the Th1 culture in co-culture with Th2 cells. This may be due to the percentage of IFNγ^+^IL-10^+^ cells not being as great as in Fig. 7a or to a greater induction of anergy in the “Th1 + IL-4” cells in Fig. 7a following a longer culture period than those in Fig. 7c. Blockade of IL-4 during co-culture restored the encephalitogenic capacity of the population of co-cultured Th1 cells. This was clearly seen in the higher disease score and the increased percentage weight loss (Fig. 7g and h).

## Discussion

Here we have investigated the influence of a classical Th2 cytokine, IL-4, on a population of self-reactive Th1 cells. As Th1 cells are repeatedly stimulated, IL-10 production increases leading to a self-regulatory phenotype^3^. In the context of repeated stimulation both *in vitro* and *in vivo*, inhibition of IL-4 signalling, through antibody blocked or IL-4Rα knockout, inhibited IL-10 production, suggesting that in this model IL-4 is the key a major cytokine for IL-10 production in Th1 cultures. The addition of either exogenously added IL-4, or Th2-derived IL-4 enhanced the production of IL-10 with no decrease in the proportion of T-bet^+^ cells. Therefore, a Th2 cytokine can modulate Th1 phenotype through the production of the anti-inflammatory cytokine IL-10. IL-4 reduced the pathogenic potential of Th1 cultures seen through a reduction in adoptively transferred EAE.

A number of cytokines upregulate IL-10 in Th1 cells notably through STAT and SMAD proteins downstream of the cytokine receptors^24^. Indeed, IL-12 has been shown to cause upregulation of IL-10 from human and murine Th1 cells *in vitro* through STAT4^10, 25^. IL-27-deficient mice infected with *T. gondii* expressed fewer co-producing IL-10^+^IFN-γ^+^ cells than wild-type animals^12^. IL-27 promotes IL-10 signalling through both STAT1^11^ and STAT3^12^. IL-21 also induces IL-10 expression in Th1 cells through STAT3 signalling^13^. TGF-β signals through SMAD4 in Th1 cells^26^ and SMAD3 in Th2 cells^27^ to positively regulate IL-10 production.

Our data adds IL-4 to the list of key cytokines that promote IL-10 production by CD4^+^ cells. IL-4 is associated with IL-10 production in Th2 cells: CD4^+^-specific IL-4Rα^−/−^ BALB/c mice exhibit a reduction in IL-10 T cell secretion in response to *Leishmania major* ^28^; IL-4R signalling activates STAT6 and GATA3 which remodel the *il10* gene locus^29, 30^. In the induction of EAE, IL-4Rα^−/−^ mice have a reduced level of IL-10 suggesting a potential IL-4 driven enhancement of IL-10 production from T cell subsets other than Th2 cells^31^. In our study, IL-4 induced IL-10 production from CD4^+^ Th1 cultures.

We have shown that cultures of polarised Th1 cells retain the mechanisms required in the initial response to IL-4, notably expression of IL-4R and the ability to phosphorylate STAT6. Initially expressed on naïve cells, IL-4R is upregulated under IL-4 stimulation^32^. In differentiating Th1 cells, under the influence of IL-12, IL-4R expression is downregulated by the transcription factor Hlx which itself is regulated by T-bet^33^. However, this does not completely abrogate IL-4R expression^34^. Our findings show that IL-4R can be re-expressed in a population of Th1 cells in response to exogenous IL-4 or repetitive stimulation leading to functional downstream signalling through STAT6, a potential mechanism by which IL-4 promotes IL-10 expression. In addition to promoting an anti-inflammatory cytokine, IL-4 also affected the functionality of Th1 cultures by reducing their encephalitogenic capacity *in vivo*. This suggests that IL-4 contributes to the dampening of a pathogenic immune response by Th1 cells through the upregulation of IL-10, a mechanism of self-regulation^3^.

The flexibility observed here in the cytokine production and transcription factor profile of cultured Th1 cells is consistent with work emphasising the plasticity of T cell subsets. Recent data has challenged the notion that T cells are restricted to a particular fate and found that cytokine expression is not as stable as previously thought. Indeed, Th1 cells can produce IL-13 under chronic stimulation, in the presence of TGF-β whereas, in lung fibrosis, Th2 cells can produce IL-9^35^. This flexibility is also displayed in other T cell subsets: the Th17 phenotype is relatively unstable and under the right conditions these cells have been shown to produce IL-9, IFN-γ and IL-22^36, 37^ and evidence that Treg cells can express transcription factors from other T cell lineages is emerging^18^. Of particular interest, multiple cell subsets have been shown to produce IL-10 including Th1, Th2, Treg and Th17 cells^1^.

The upregulation of IL-10 by Th1 cultures under chronic stimulation seen over repeated rounds of stimulation both *in vitro* and *in vivo* demonstrates the degree of plasticity within this T cell lineage. This correlates with work demonstrating that a high dose of antigen upregulates IL-10 in Th1 cells both *in vitro* ^10^ and *in vivo* ^19^ and presents a mechanism of self-regulation in Th1 cells^3^. Here we present *in vitro* data showing that chronic stimulation of differentiated Th1 cultures leads to the expression of IL-4 that in turn enhances the upregulation of IL-10. Polarised Th2 cells, rather than inhibiting the Th1 phenotype, contribute to the dampening down of an excessive Th1 response by inducing the anti-inflammatory cytokine IL-10. Indeed, we observed the maintenance of T-bet in Th1 cultures with addition of exogenous IL-4 or co-cultured with Th2 cells alongside the induction of IL-10.

The inhibition of the upregulation of IL-10 expression through blocking IL-4 was more effective during co-culture of Th1 and Th2 cells separated by a semi-permeable membrane of a transwell. In this system, it is likely that Th2-derived IL-4 is more diffuse and as such the IL-4 blocking antibody is better able to inhibit it. This also points to a potential need for the interaction of the Th1 and Th2 cells with the formation of a synapse between the two T cell subsets, allowing a directed secretion of IL-4. The concept of T-T cell contact dependent mechanisms for regulation has been demonstrated in a Treg context^38, 39^, for directional secretion of IFN-γ between CD8^+^ cells^40^ and IL-2 between CD4^+^ T cells^41^.

The cross-regulation between Th1 and Th2 cells is mediated, in part, by the transcription factors that they express. Here we observed the co-expression of T-bet and c-Maf either in the population of Th1 cells co-cultured with Th2 cells or restimulated Th1 cultures. This points to a transcriptional regulation leading to a change in the cytokine profile. Blockade of IL-4 inhibited c-Maf expression suggesting that c-Maf may be downstream of IL-4R signalling and represent a mechanism by which IL-4 influences transcription and thus IL-10 production in populations of Th1 cells. c-Maf regulates IL-4^42^ and also IL-10 expression in macrophages^14^ as well as CD4^+^ T cells^10, 17, 43^. However, IL-4 blockade does not completely inhibit c-Maf expression suggesting that IL-4 is not the sole regulator of c-Maf. For example, in human CD4^+^ T cells, IL-2 is a regulator of c-Maf^44^. When thinking about plasticity in T cell lineages, it has been suggested that we should think in terms of gradients of transcription factors within a cell^45^. Therefore, in Th2 cell regulation of Th1 cells, a gradient of c-Maf upregulation in Th1 cells may indicate how likely it is to upregulate expression of IL-10.

Interestingly, epidemiological evidence shows a rise in autoimmune diseases in developed countries; furthermore, there is an inverse relationship between Th1 driven autoimmune diseases and Th2 helminth infections suggesting protective effects of helminths^46^. Indeed, amelioration of inflammatory bowel disease and type I diabetes has been observed with helminth infection^47, 48^. The use of helminths to treat multiple sclerosis is currently in clinical trials with upregulation of IL-4 and IL-10 seen in serum^49^. IL-4 has also been observed in the recovery from EAE^50^. Several studies have reported an amelioration of EAE when mice are either pre-treated or infected during disease with helminths or protozoans^51, 52^. In the case of infection with protozoans, these decreased effects on disease severity were due to IL-10^52^. An immunoregulatory environment is created by a Th2 response and we propose that IL-4 in these contexts is enhancing IL-10.

Flexibility in T cell subsets is advantageous especially as T cells migrate and encounter many different cues. Plasticity has evolved so that the immune system can respond to the changing environment and effectively control diverse infections. We conclude that IL-4 plays a critical role in the self-regulation of Th1 pathology by upregulation of IL-10. This opens new avenues for therapeutic intervention in Th1 driven diseases.

## Methods

### Mice

All murine strains were bred in-house onto the H-2^u^ haplotype background under specific pathogen-free conditions at the University of Bristol. B10.PL (H-2^u^) mice were originally purchased from The Jackson Laboratory. The generation of Tg4 strain expressing the Vβ8.2 TCR specific for MBP Ac1-9 has previously been described^53^. Tg4 CD45.1 were a kind gift from Steve Anderton, Edinburgh. IL-4 receptor alpha (IL-4Rα) deficient C57BL/6 mice (a kind gift from Judith Allen, Edinburgh) were crossed with Tg4 mice to generate Tg4^+/+^IL-4Rα^−/−^ mice. Expression of the transgenic Vβ8 TCR, H-2^u^ was confirmed by flow cytometry and deletion of IL-4Rα was assessed by PCR of genomic DNA isolated from tail or ear tissue to determine the lack of exons 7, 8 and 9 of IL-4Rα gene. Male and female mice aged 6–12 weeks were used and were equally distributed between groups based on age and sex. All experiments were carried out according to animal welfare codes directed by the University of Bristol ethical review committee, under the regulation of the UK Home Office Project Licence number 30/2705 held by D.C.Wraith.

### Antibodies, cytokines and other reagents

The acetylated N-terminal peptides of myelin basic protein, MBPAc1-9 [4A] (AcASQARPSQR), MBPAc1-9 [4K] (AcASQKRPSQR) and [4Y] (AcASQYRPSQR) were synthesized by GL Biochem Shanghai. *In vitro* stimulations and assays were performed in complete RPMI (Lonza). All flow cytometric staining was performed in fluorescent activated cell sorting (FACS) buffer. A list of antibodies and cytokines and details of their use in this study can be found in Table 1.

**Table 1:** Antibodies and cytokines.

Epitope	Clone	Conjugation	Supplier	Working concentration
CD4	GK1.5	Alexa700	Biolegend	2 μg/ml
CD45.1	A20	FITC	eBioscience	2 μg/ml
IL-10	ES5-16E3	APC	eBioscience	1 μg/ml
IFNγ	XMG1.2	PerCP-Cy5.5	eBioscience	0.5 μg/ml
IL-4	11B11	PE	eBioscience	0.5 μg ml
IL-17A	TC11-18H10.1	PE	eBioscience	1 μg/ml
IL-2	JES6-5H4	eFluor450	eBioscience	2 μg/ml
cMaf	SYMOF1	eFluor660	eBioscience	2 μg/ml
T-bet	4B10	eFluor660	eBioscience	2 μg/ml
Phospho-STAT6	CHI2S4N	PerCP-eFlour610	eBioscience	2 μg/ml
CD3ε	2C11	None	eBioscience	1 μg/ml
CD28	37.51	None	eBioscience	2 μg/ml
IL-4	11B11	None	BioXCell	10 μg/ml
IFN-γ	XMG1.2	None	BioXCell	10 μg/ml
rmIL-12	None	None	Peprotech	5 ng/ml
rmIL-4	None	None	Peprotech	10 ng/ml
rhIL-2	None	None	R&D Sytems	20 U/ml

APC – allophycocyanin.

FITC - fluorescein isothiocyanate.

PE – phycoerythrin.

PerCP - peridinin chlorophyll protein complex.

### Cell isolation

Spleens were disaggregated and red blood cells removed by osmotic lysis. Where indicated, CD4^+^ T cells were isolated using negative magnetic separation with CD4^+^ T cell Isolation Kit II (Miltenyi Biotech) or MagniSort™ Mouse CD4^+^ T cell Enrichment Kit (eBioscience). Following culture, live cells were isolated using Ficoll centrifugation (Ficoll-Paque^TM^ Premium 1.084 g/ml; GE Healthcare).

### *In vitro* repeated stimulation

All cells were cultured in humidified incubators at 37 °C, 5% CO_2_ at a concentration of 2.5 × 10^6^ cells per well in 24 well flat-bottomed tissue culture plates (Corning, Artington, UK). The primary stimulation of the cells corresponds to Th1 polarisation of Tg4 splenocytes with 10 μg/ml MBP Ac1-9[4K] using rmIL-12 and anti-IL-4. On day 7 of culture, polarised live cells were isolated using Ficoll centrifugation. These cells were subsequently stimulated for a second time (secondary stimulation) with sex-matched irradiated (2500 rad using a ^137^caesium γ-radiation source) B10.PL as APCs (at a ratio of 1:2T cell:APC) and 5 μg/ml MBP Ac1-9[4Y] under the conditions indicated in each experiment. On day 7 of culture, live cells were isolated using Ficoll centrifugation and stimulated for a third time (tertiary stimulation) under the same conditions as the secondary stimulation. Cells were cultured for a further 7 days. For secondary and tertiary stimulation in an APC-independent environment, CD4^+^ live cells from the primary stimulation of Th1 polarisation were isolated by Ficoll centrifugation and magnetic separation. The secondary stimulation was performed using anti-CD3 and anti-CD28 coated plates under the conditions indicated in the different experiments and cells cultured for 7 days. The tertiary stimulation was performed with live CD4^+^ T cells isolated and cultured under the same conditions as the secondary stimulation. In all the cycles of stimulation under the different stimulations, cells were split on day 3 of culture with the addition of 20 U/ml rhIL-2 and were subsequently split as required.

### Th1-Th2 co-cultures and transwell assays

Th2 cells were polarised from Tg4 splenocytes with 10 μg/ml MBP Ac1-9[4K] using rmIL-4 and anti-IFN-γ. Cells were split on day 3 of culture with the addition of 20U/ml rhIL-2 and then were subsequently split as needed. Following a 7 day culture, live Th2 cells were co-cultured with polarised Th1 cells at a ratio of 1:1 Th1:Th2 and stimulated sex-matched irradiated B10.PL splenocytes as APCs at a ratio of 1:2 T cell:APC and 5 μg/ml MBP Ac1-9[4Y]. Ratios were calculated for a total of 3 × 10^6^ cells per well of a 24 well culture plate. For transwell co-cultures, transwell inserts of 6.5mm diameter with 0.4μm pore size (Corning Incorporated Costar) were used. Th2 were cultured in the top chamber and Th1 in the bottom chamber. Sex-matched irradiated B10.PL splenocytes as APCs were placed on both sides of the membrane.

### Flow cytometric staining

Surface and intracellular cytokine staining was performed following a 3-hour stimulation in culture medium containing 5 ng/ml phorbol 12-myristate 13-acetate (PMA), 500 ng/ml ionomycin (both Sigma-Aldrich) and 0.57 μl/ml GolgiStop (BD Biosciences). Cells were stained with Fixable Viability Dye eFluor® 780 (eBioscience) prior to surface staining. Cytokine staining was performed using Intracellular Fixation Buffer and Permeabilization Buffer (eBioscience). Intranuclear staining (for T-bet or cMaf) was performed using FoxP3 Staining Buffers (eBioscience). Data was acquired on an LSR-II or Fortessa X-20 cytometer (Becton Dickinson) using FACSDiva software and analysed using FlowJo software (Treestar Inc.). Live CD4^+^ T cell gating strategy is shown in Supplementary Fig. S1.

### Measurement of STAT6 activation

Cells were stimulated for 20 minutes at 37 °C with IL-4. Cells were washed in cold FACS buffer and surface staining was performed on ice. Cells were fixed on ice in 2% paraformalhyde and washed in cold FACS buffer. Cells were permeabilised in 90% methanol diluted in PBS for an hour or overnight at −20 °C. Cells were stained for phoso-STAT6 in FACS buffer at room temperature for 1 hour.

### Enzyme-linked immunosorbent assay (ELISA)

Cytokine protein levels were measured using ELISA with paired antibodies according to the manufacturer’s instructions (BD Biosciences). Optical densities were read at 450/595 nm on a Spectramax190 microplate reader and analysed using SoftMax Pro software (Molecular Devices).

### EAE induction and evaluation

EAE was induced in Tg4 mice on day 0 by intraperitoneal of 1 ×﻿﻿ 10^7^ live Th1 cells following their third stimulation or following co-culture with Th2 cells under the conditions indicated in each experiment. The animal’s baseline body weight was recorded and mice were weighed and monitored daily for clinical signs of EAE. Animals were graded according to the following scoring system: 0, no disease; 1, flaccid tail; 2, hindlimb weaknesss and/or impaired righting reflex; 3, total hindlimb paralysis; 4, hind and forelimb paralysis; 5, moribund.

### Histopathology

Hematoxylin and eosin (H&E) staining was carried out on dissected spinal cords from mice following EAE induction by adoptive transfer of Th1 cells. These were fixed with 4% paraformaldehyde, embedded in paraffin and sectioned longitudinally. Analysis was performed on a light microscope and carried out in a blinded fashion. Demyelination analysis was done by luxol fast blue/cresyl. Sections were assessed using the following scoring system: 0, histologically normal; 1, a few discrete perivascular cuffs with no significant demyelination; 2, numerous discrete small to medium perivascular cuffs affecting parenchyma and meninges. May be focal demyelination of individual axons associated with mild extension of cuff to surrounding parenchyma; 3, numerous medium to large perivascular cuffs may coalesce and extend significantly into parenchyma. Meninges also involved. May be demyelination of axonal groups associated with extension into parenchyma.

### Data availability

All data generated or analysed during this study are included in this published article (and its Supplementary Information files).

## Electronic supplementary material

Supplementary Information
