# Supplementary material for: IL-4 enhances IL-10 production in Th1 cells: implications for Th1 and Th2 regulation
Source: Sci Rep. 2017 Sep 12;7:11315. doi: 10.1038/s41598-017-11803-y (PMC5595963; doi:10.1038/s41598-017-11803-y)
Supplement: Supplementary file 1 — Supplementary Information [file 41598_2017_11803_MOESM1_ESM.pdf]

IL-4 enhances IL-10 production in Th1 cells: implications for Th1 and Th2 regulation

Ruth E. Mitchell[1;2], Masriana Hassan[1;3], Bronwen R. Burton[1;4], Graham Britton[1;5], Elaine V. Hill[1], Johan Verhagen[1;6], David C. Wraith[1;7] *

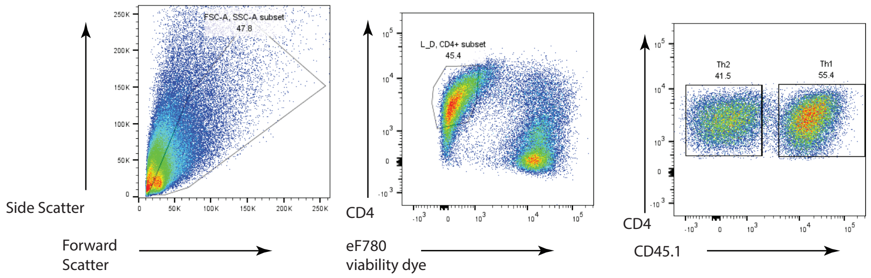

<u>Supplementary Figure S1: CD4+ T cell gating strategy</u>

An example is shown of the FACS gating strategy used. Lymphocytes were gated in a FSC/SSC plot as indicated and live $CD4^+$ cells within this population were gated in a CD4-Alexa700 / ef780 viability dye plot as indicated. If in a co-culture of Th1 and Th2 cells, cells were gated on $CD45.1^+$ cells to identify the Th1 cells. These cells were then further analysed for intracellular cytokines or transcription factors.

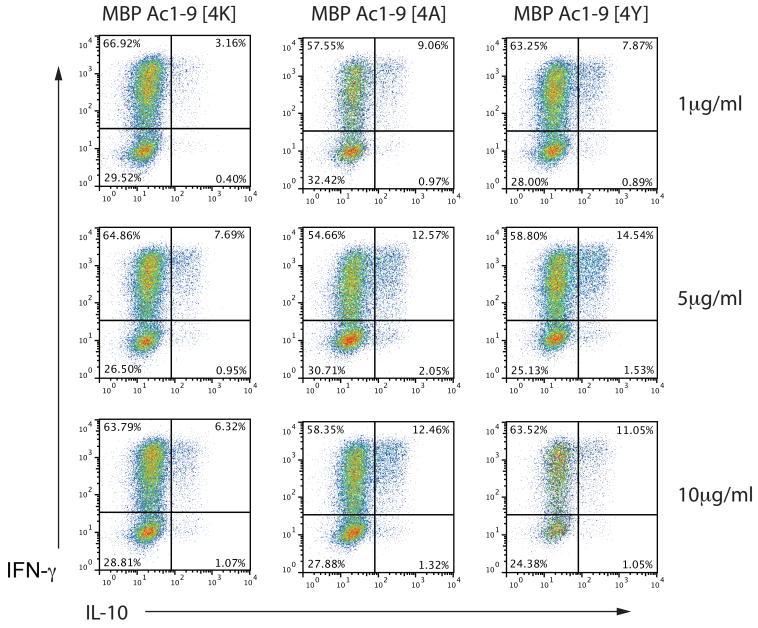

<u>Supplementary Figure S2: The affinity and dose of peptide determine the differentiation of IL-10 secreting CD4+ cells</u>

Splenic Tg4 CD4+ T cells were cultured in the presence of irradiated B10.PL splenocytes as APCs and MBP Ac1-9 peptide affinity variants (MBP Ac1-9[4A], MBP Ac1-9[4K], MBP Ac1-9[4Y]) at a number of different concentrations (1µg, 5µg, 10µg). Intracellular cytokine staining was carried out on day 7 of the second stimulation, following PMA and ionomycin stimulation. FACS plots from a single experiment are gated on live CD4+ cells and show IFN-γ, IL-4 and IL-10 expression.
